# Supplementary material for: A Global Evaluation of the Performance Indicators of Colorectal Cancer Screening with Fecal Immunochemical Tests and Colonoscopy: A Systematic Review and Meta-Analysis
Source: Cancers (Basel). 2022 Feb 21;14(4):1073. doi: 10.3390/cancers14041073 (PMC8870403; doi:10.3390/cancers14041073)
Supplement: Supplementary file 1 [file cancers-14-01073-s001.zip › cancers-1581261-supplementary.pdf]

Table S1. Search strategy for article selection.

Database	Search Strategy	Result
PubMed	(colorectal OR bowel OR colon OR rectal) AND (cancer OR tumor OR neoplasm OR carcinoma) AND (Screening Program* OR screening programme* OR "early detection of cancer" OR "mass screening" OR "organized screening" OR "organised screening" OR "population-based screening" OR "general screening" OR "community-based screening") AND (Fecal occult blood test* OR Faecal occult blood test* OR Fecal immunochemical test* OR Faecal immunochemical test* OR Colonoscopy OR Endoscopy) AND (Performance OR Uptake rate* OR participation rate* OR compliance rate* OR adherence rate* OR Inadequate rate* OR Invalid rate* OR Positivity rate* OR Negative rate* OR Accuracy OR "Positive Predictive Value" OR "Negative Predictive Value" OR sensitivity OR specificity OR detection rate* OR Complete colonoscopy rate* OR Complication rate* OR Cecal intubation rate* OR "Colonoscope withdrawal time" OR "Quality of bowel preparation")	2784
Ovid Medline	(colorectal OR bowel OR colon OR rectal) AND (cancer OR tumor OR neoplasm OR carcinoma) AND (Screening Program OR screening programme OR early detection of cancer OR mass screening OR population-based screening OR general screening OR community-based screening OR organized screening OR organised screening) AND (Fecal occult blood test* OR Fecal immunochemical test* OR Colonoscopy OR Endoscopy) AND (Performance OR Uptake rate* OR participation rate* OR compliance rate* OR adherence rate* OR Inadequate rate* OR Invalid rate* OR Positivity rate* OR Negative rate* OR Accuracy OR Positive Predictive Value OR PPV OR Negative Predictive Value OR NPV OR sensitivity OR specificity OR detection rate* OR Complete colonoscopy rate* OR Complication rate* OR Cecal intubation rate* OR Colonoscope withdrawal time OR Quality of bowel preparation)	2106
Cochrane	(colorectal OR bowel OR colon OR rectal) AND (cancer OR tumor OR neoplasm OR carcinoma) AND ("screening program" OR "screening programs" OR "screening programes" OR "screening programme" OR "screening programmes" OR "early detection of cancer" OR "organized screening" OR "organised screening" OR "mass screening" OR "population-based screening" OR "general screening" OR "community-based screening") AND ("fecal occult blood test" OR "fecal occult blood testing" OR "fecal occult blood testings" OR "fecal occult blood tests" OR "faecal occult blood test" OR "faecal occult blood testing" OR "faecal occult blood tests" OR "fecal immunochemical test" OR "fecal immunochemical testing" OR "fecal immunochemical tests" OR "faecal immunochemical test" OR "faecal immunochemical testing" OR "faecal immunochemical tests" OR colonoscopy OR endoscopy) AND (Performance OR "uptake rate" OR "uptake rates" OR "participation rate" OR "participation rates" OR "compliance rate" OR "compliance rates" OR "adherence rate" OR "adherence rates" OR "inadequate rate" OR "inadequate rates" OR "invalid rate" OR "invalid rates" OR "positivity rate" OR "positivity rates" OR "negative rate" OR "negative rates" OR Accuracy OR "Positive Predictive Value" OR "Negative Predictive Value" OR sensitivity OR specificity OR "detection rate" OR "detection rates" OR "Complete colonoscopy rate" OR "Complete colonoscopy rates" OR "complication rate" OR "complication rates" OR "cecal intubation rate" OR "cecal intubation rates" OR "Colonoscope withdrawal time" OR "Quality of bowel preparation")	480
EMBase	('colorectal' OR 'bowel' OR 'bowel'/exp OR 'colon' OR 'colon'/exp OR 'rectal') AND ('cancer' OR 'cancer'/exp OR 'tumor' OR 'tumor'/exp OR 'neoplasm' OR 'neoplasm'/exp OR 'carcinoma' OR 'carcinoma'/exp)<br>AND ('screening program' OR 'screening program'/exp OR 'screening programs' OR 'screening programes' OR 'screening programme' OR 'screening programme'/exp OR 'screening programmes' OR 'early detection of cancer' OR 'early detection of cancer'/exp OR 'organized screening' OR 'organised screening' OR 'mass screening' OR 'mass screening'/exp OR 'population-based screening' OR 'general screening' OR 'community-based screening')<br>AND ('fecal occult blood test' OR 'fecal occult blood test'/exp OR 'fecal occult blood testing' OR 'fecal occult blood testing'/exp OR 'fecal occult blood testings' OR 'fecal occult blood tests' OR 'faecal occult blood test' OR 'faecal occult blood testing' OR 'faecal occult blood tests' OR 'fecal immunochemical test' OR 'fecal immunochemical test'/exp OR 'fecal immunochemical testing' OR 'fecal immunochemical testing'/exp OR 'fecal immunochemical tests' OR 'faecal immunochemical test' OR 'faecal immunochemical testing' OR 'faecal immunochemical tests' OR 'colonoscopy'/exp OR colonoscopy OR 'endoscopy'/exp OR endoscopy)<br>AND (Performance OR 'Performance'/exp OR 'uptake rate' OR 'uptake rates' OR 'participation rate' OR 'participation rates' OR 'compliance rate' OR 'compliance rates' OR 'adherence rate' OR 'adherence rates' OR 'inadequate rate' OR 'inadequate rates' OR 'invalid rate' OR 'invalid rates' OR 'positivity rate' OR 'positivity rates' OR 'negative rate' OR 'negative rates' OR Accuracy OR 'Accuracy'/exp OR 'Positive Predictive Value' OR 'Positive Predictive Value'/exp OR 'Negative Predictive Value' OR 'Negative Predictive Value'/exp OR sensitivity OR 'sensitivity'/exp OR specificity OR 'specificity'/exp OR 'detection rate' OR 'detection rates' OR 'Complete colonoscopy rate' OR 'Complete colonoscopy rates' OR 'complication rate' OR 'complication rate'/exp OR 'complication rates' OR 'cecal intubation rate' OR 'cecal intubation rate'/exp OR 'cecal intubation rates' OR 'Colonoscope withdrawal time' OR 'Quality of bowel preparation')	6883

Table S2. Characteristics of included studies.

Included Articles	Country	Region/City	Sample Size	Project Period	Age Range	Round	FIT Number	Cut-off Value *
Abu Hassan, M. R. 2016 [1]	Malaysia	Kedah	750	2013	50-	Initial	2	-
Auge, J. 2014 [2]	Spain	Barcelona	197,839	2009–2012	50–69	Initial	1	100 ng/mL
Bankovic Lazarevic, D. 2016 [3]	Serbia	-	99,592	2013–2014	50–74	Initial	1	-
Binefa, G. 2016 [4]	Spain	Hospitalet de Llobregat	76,824	Feb 2000–Nov 2012	50–69	Subsequent	1	100 ng/mL
Blom, J. 2019 [5]	Sweden	Stockholm-Gotland	87,269	Oct 2015–Sep 2016	60–69	Subsequent	1	200 ng/mL (women), 400 ng/mL (men)

Bujanda, L. 2015 [6]	Spain	Basque country	463,927	Apr 2009–Apr 2013	50–69	Total	1	100 ng/mL
Buron, A. 2017 [7]	Spain	Barcelona	172,962	Dec 2009–Dec 2011	50–69	Initial	1	100 ng/mL
Buttigieg, G. 2018 [8]	Malta	-	14,287	Jan–Dec2016	59–61	Initial	-	-
Chou, C. K. 2016 [9]	China	Taiwan	3,363,896	Jan 2004–Dec 2013	50–69	Subsequent	1	100, 8 & 12 ng/mL
Ciatto, S. 2007 [10]	Italy	Florence	81,047	Dec 1996–Sep 2005	48–76	Subsequent	1	100 ng/mL
Clark, G. 2020 [11]	UK	Scotland	919,665	Nov 2017–Oct 2018	50–74	Subsequent	1	400 ng/mL
Crotta, S. 2004 [12]	Italy	Chatillon and St Vincent	2961	Sep 2001–Dec 2001	50–74	Initial	1	100 ng/mL
Denters, M. J. 2012 [13]	Netherlands	Amsterdam	11,234	Jun 2006–Feb 2007	50–74	Total	1	50 ng/mL
Fenocchi, E. 2006 [14]	Uruguay	Montevideo	11,734	Jun 1997–Jul 2004	50-	Initial	1	100 ng/mL
Grazzini, G. 2000 [15]	Italy	Florence	15,235	Oct 1996–Jul 1998	50–70	Initial	1	-
Grazzini, G. 2004 [16]	Italy	Tuscany	192,583	Jun 2000–Dec 2001	50–70	Initial	1	100 ng/mL (80 ng/mL for one site)
Grazzini, G. 2009 [17]	Italy	Northern & Central	36,646	Sep 2005–Jun 2007	50–69	Initial	2	80 ng/mL
Guo, F. 2020 [18]	Belgium	Wallonia and Brussels	28,217	2014–2017	50–74	Subsequent	1	75 ng/mL
Hoeck, S. 2019 [19]	Belgium	Flanders	1,184,462	2013–2014	56–74	Initial	-	-
Kapidzic, A. 2015 [20]	Netherlands	Rotterdam	17,808	Nov 2006–Dec 2010	50–74	Total	1	50 ng/mL
Khuhaprema, T. 2014 [21]	Thailand	Lampang	127,301	Apr 2011–Nov 2012	50–65	Initial	1	200 ng/mL
Kim, D. H. 2018 [22]	Korea	-	51,439	2009–2015	50-	Subsequent	1	100 ng/mL
Koïvogui, A. 2018 [23]	France	Seine-Saint-Denis	132,473	2015–Dec 2016	50–74	Subsequent	1	150 ng/mL
Launoy, G. D. 2005 [24]	France	Cotentin	7421	Jan 2001–Dec 2002	50–74	Initial	2	20 ng/mL
Lund, M. 2019 [25]	Denmark	Central Denmark Region	8256	Jul 2015–Jun 2017	50–74	Subsequent	1	100 ng/mL
Manzano-Robleda, M. D. C. 2020 [26]	Mexico	Mexico City	892	Oct 2017–Jan 2019	50–74	Initial	1	20 ng/mL
McNamara, D. 2011 [27]	Ireland	-	9993	2008–2010	50–74	Initial	2	100 ng/mL
Miuțescu, B. 2013 [28]	Romania	Timisoara	1111	-	50–74	Initial	-	-
Moss, S. 2017 [29]	UK	-	40,930	Apr–Oct 2014	59–75	Subsequent	1	100 ng/mL
Nakama, H. 1996 [30]	Japan	Nagano	3982	1991	40-	Initial	1	-
Nakama, H. 2001 [31]	Japan	Nagano	17,432	1994–1999	40-	Subsequent	4	-
Njor, S. H. 2018 [32]	Denmark	-	1,437,836	Mar 2014–Dec 2016	50–74	Initial	1	100 ng/mL
Okada, T. 2019 [33]	Chile	Santiago,Valparaiso,PuntaArenas, Coquimbo,Osorno,Valdivia	26,444	Jun 2012–Dec 2017	50–75	Initial	2	100 ng/mL
Parente, F. 2013 [34]	Italy	Lecco	159,702	Sep 2005–Dec 2009	50–69	Total	1	100 ng/mL
Park, M. J. 2011 [35]	Korea	-	23,756,667	Jan 2004–Dec 2009	50-	Total	-	-
Pellat, A. 2018 [36]	France	Paris	409,340	Jan 2016–Jul 2017	50–74	Subsequent	1	150 ng/mL
Piette, C. 2017 [37]	France	Ille-et-Vilaine, Brittany	162,347	May 2015–Dec 2015	50–74	Subsequent	1	150 ng/mL
Portillo, I. 2018 [38]	Spain	Basque country	1,377,398	Jan 2009–Sep 2016	50–69	Subsequent	1	100 ng/mL
Poskus, T. 2015 [39]	Lithuania	Vilnius, Kaunas, and Klaipeda	271,396	Jul 2009–Jul 2012	50–74	Initial	3, 6, 9	30, 200 and 100 ng/mL

Remes-Troche, J. M. 2020 [40]	Mexico	Veracruz	502	May 2015–Jan 2016	50–90	Initial	1	100 ng/mL
Rutka, M. 2020 [41]	Hungary	Csongrád county	22,130	Jul 2013–Jul 2015	50–70	Initial	2	100 ng/mL
Salimzadeh, H. 2017 [42]	Iran	Tehran	1542	-	45–75	Initial	1	100 ng/mL
Shahidi, N. 2016 [43]	Canada	British Colombia	168,599	Nov 2013–Dec 2014	50–74	Subsequent	1	50 ng/mL
Shim, J. I. 2010 [44]	Korea	-	4,640,365	2008	50-	Subsequent	-	-
Steele, R. J. 2013 [45]	UK	Scotland	66,225	Jul 2010–Jan 2011	50–74	Subsequent	1	400 ng/mL
Stegeman, I. 2015 [46]	Netherlands	Amsterdam	3566	Jul 2011–Jan 2012	50–75	Subsequent	1	50 ng/mL
Suchanek, S. 2014 [47]	Czech Republic	-	935,729	2009–2011	50-	Subsequent	-	-
Sultanian, R. 2020 [48]	Canada	Edmonton, Alberta	2167	Jan–Dec 2014	50–75	Subsequent	1	375 ng/mL
Teixeira, C. R. 2017 [49]	Brazil	Southern	1039	Apr 2015–Jan 2016	50–75	Initial	-	50 ng/mL
Telford, J. 2016 [50]	Canada	British Columbia	19,684	2009–2013	50–74	Initial	2	100 ng/mL
Tepeš, B. 2014 [51]	Slovenia	Ljubljana, Kranj and Celje	9091	Jun-Dec 2008	64–68	Initial	2	100 ng/mL
Tepeš, B. 2017 [52]	Slovenia	-	536,709	Apr 2009–Mar 2011	50–69	Initial	2	100 ng/mL
Triantafillidis, J. K. 2010 [53]	Greece	Athens, Salonika, Iraklion	11,089	2008–2009	55–72	Total	-	50 ng/mL or higher or 6 μg/g feces.
Vitellius, C. 2019 [54]	France	Maine-et-Loire	203,117	Jun 2015–Dec 2016	50–74	Subsequent	-	150 ng/mL
Zorzi, M. 2006 [55]	Italy	-	331,333	2004	50–69	Subsequent	1	-
Zorzi, M. 2007 [56]	Italy	-	827,473	2005	50–69	Subsequent	1	100 ng/mL
Zorzi, M. 2008 [57]	Italy	-	2,106,916	2006	50–69	Subsequent	1	100 ng/mL
Zorzi, M. 2009 [58]	Italy	-	2,584,833	2007	50–69	Subsequent	1	80,100 ng/mL
Zorzi, M. 2010 [59]	Italy	-	2,593,411	2008	50–69	Subsequent	1	100 ng/mL
Zorzi, M. 2011 [60]	Italy	-	2,952,892	2009	50–69	Subsequent	1	100 ng/mL
Zorzi, M. 2015_1 [61]	Italy	-	7,744,295	2011–2012	50–69	Subsequent	1	80,100 ng/mL
Zorzi, M. 2015_2 [62]	Italy	-	75,596	2010	50–69	Subsequent	1	100 ng/mL
Zorzi, M. 2018 [63]	Italy	Veneto	178,828	2002–2015	50–69	Subsequent	1	100 ng/mL
			**Reports**					
Australia. 2008 [64]	Australia	-	1,010,073	Aug 2006–Jun 2008	55–74	Total	2	-
Australia. 2012 [65]	Australia	-	2,097,520	Jul 2008–Jun 2011	50–65	Subsequent	2	-
Australia. 2013 [66]	Australia	-	929,433	Jul 2011–Jun 2012	50–65	Subsequent	2	-
Australia. 2014 [67]	Australia	-	963,518	Jul 2012–Jun 2013	49–67	Subsequent	2	-
Australia. 2015 [68]	Australia	-	1,415,555	Jul 2013–Jun 2014	49–67	Subsequent	2	-
Australia. 2017 [69]	Australia	-	2,608,599	2014–2015	50–74	Subsequent	2	-
Australia. 2019 [70]	Australia	-	4,100,498	2016–2017	50–74	Subsequent	2	-
Australia. 2020 [71]	Australia	-	5,074,980	2018	50–74	Subsequent	2	-
Canada. 2013 [72]	Canada	-	-	Jan 2009–Dec 2011	50–74	-	1+2	100 ng/mL
Canada. 2014 [73]	Canada	-	-	2011–2012	50–74	-	-	100, 300 ng/mL

Canada. 2017 [74]	Canada	-	-	Jan 2013–Dec 2014	50–74	-	1+2	50, 100 ng/mL
Hong Kong. 2019 [75]	China	Hong Kong	159,000	Sep 2016–Sep 2019	56–75	Total	-	100 ng/mL
Ireland. 2016 [76]	Ireland	-	488,628	2012–2015	55–74	Initial	-	100 ng/mL
Ireland. 2019 [77]	Ireland	-	437,001	2016–2017	60–69	Subsequent	-	-
Netherlands. 2015 [78]	Netherlands	-	189,610	2014	55–75	Initial	1	88, 275 ng/mL
Netherlands. 2016 [79]	Netherlands	-	1,169,391	2015	55–75	Subsequent	1	275 ng/mL
Netherlands. 2017 [80]	Netherlands	-	1,457,976	2016	55–75	Subsequent	1	275 ng/mL
Netherlands. 2018 [81]	Netherlands	-	1,941,121	2017	55–75	Subsequent	1	275 ng/mL
Netherlands. 2019 [82]	Netherlands	-	2,348,534	2018	55–75	Subsequent	1	275 ng/mL
New Zealand. 2017 [83]	New Zealand	-	121,567	Jan 2012–Dec 2015	50–74	Total	-	-
UK. 2014 [84]	UK	North Ireland	-	Apr 2010–Mar 2013	60–71	Total	-	-
UK. 2019 [85]	UK	Scotland	1,866,332	Nov 2017–Oct 2018	50–74	Subsequent	-	-

* The cut-off of 100 ng hemoglobin per mL of buffer solution was corresponding to 20 μg hemoglobin/g feces. FIT: fecal immunochemical tests.

Table S3. The Appraisal of cross-sectional studies (AXIS) of included manuscripts.

Included Manuscripts	1	2	3	4	5	6	7	10	11	12	13	14	15	16	17	18	19	20
Abu Hassan, M. R. 2016 [1]	1	1	0	1	0	1	0	0	0	1	0	0	1	1	1	1	9	1
Auge, J. 2014 [2]	1	1	1	1	0	1	0	1	1	0	0	0	1	1	1	1	0	9
Bankovic Lazarevic, D. 2016 [3]	1	1	1	1	1	1	0	0	1	0	0	0	1	1	1	0	9	9
Binefa, G. 2016 [4]	1	1	1	1	1	1	0	1	1	0	0	0	1	1	1	1	0	1
Blom, J. 2019 [5]	1	1	1	1	1	1	0	1	1	1	0	0	1	1	1	1	0	1
Bujanda, L. 2015 [6]	1	1	1	1	1	1	0	0	1	1	0	0	1	1	1	1	0	9
Buron, A. 2017 [7]	1	1	1	1	1	1	1	1	1	1	1	1	1	1	1	1	0	1
Buttigieg, G. 2018 [8]	1	1	0	0	0	0	0	0	0	1	0	0	1	1	1	1	9	0
Chou, C. K. 2016 [9]	1	1	1	1	1	0	0	1	1	1	0	0	1	1	1	1	0	1
Ciatto, S. 2007 [10]	1	1	1	1	1	1	0	1	1	1	0	0	1	1	1	0	9	9
Clark, G. 2020 [11]	1	1	1	1	1	1	0	0	1	1	0	0	1	1	1	1	0	1
Crotta, S. 2004 [12]	1	1	0	1	1	0	0	0	0	1	0	0	1	1	1	1	0	1
Denters, M. J. 2012 [13]	1	1	0	1	1	1	1	0	1	1	0	0	1	1	1	0	0	1
Fenocchi, E. 2006 [14]	1	1	0	1	9	1	0	0	1	1	0	0	1	1	1	0	0	1
Grazzini, G. 2000 [15]	1	1	0	1	1	1	0	0	0	1	0	0	1	1	1	0	0	9
Grazzini, G. 2004 [16]	1	1	1	1	1	1	1	0	0	0	0	0	1	1	1	1	9	9
Grazzini, G. 2009 [17]	1	1	0	1	1	1	0	1	1	1	0	0	1	1	1	0	9	1
Guo, F. 2020 [18]	1	1	1	1	1	1	0	1	1	1	0	0	1	1	1	1	0	1
Hoeck, S. 2019 [19]	1	1	1	1	0	0	0	1	1	1	0	1	1	1	1	1	0	1
Kapidzic, A. 2015 [20]	1	1	1	1	1	1	0	1	1	0	0	0	1	1	1	1	0	1
Khuhaprema, T. 2014 [21]	1	1	1	1	1	1	0	0	1	1	0	0	1	1	1	1	0	1
Kim, D. H. 2018 [22]	1	1	1	1	0	1	0	1	1	1	0	0	1	1	1	1	0	1
Koïvogui, A. 2018 [23]	1	1	1	1	1	1	0	1	1	1	0	0	1	1	1	1	0	1
Launoy, G. D. 2005 [24]	1	1	0	1	0	0	0	0	1	1	0	0	1	1	1	1	9	9
Lund, M. 2019 [25]	1	1	0	1	1	1	0	1	1	0	0	0	1	1	1	1	0	1
Manzano-Robleda, M. D. C. 2020 [26]	1	1	0	1	1	1	0	0	1	1	1	1	1	1	1	1	0	1
McNamara, D. 2011 [27]	1	1	0	1	1	0	0	0	0	0	0	0	1	1	1	0	9	9
Miuțescu, B. 2013 [28]	1	1	0	1	0	1	0	1	1	1	0	0	1	1	1	0	9	1
Moss, S. 2017 [29]	1	1	1	1	1	0	1	1	1	1	0	1	1	1	1	0	0	9
Nakama, H. 1996 [30]	1	1	0	1	0	0	0	0	0	1	0	0	1	1	1	1	9	9
Nakama, H. 2001 [31]	1	1	0	1	0	0	0	1	1	1	0	0	1	1	1	0	9	9
Njor, S. H. 2018 [32]	1	1	1	1	1	1	0	0	0	1	0	0	1	1	1	1	0	9

Okada, T. 2019 [33]	1	1	0	1	1	1	0	1	1	1	0	0	1	1	1	1	0	1
Parente, F. 2013 [34]	1	1	1	1	1	1	1	1	1	0	0	0	1	1	1	0	0	9
Park, M. J. 2011 [35]	1	1	1	1	9	9	0	0	0	1	0	0	1	1	1	0	9	9
Pellat, A. 2018 [36]	1	1	1	1	1	1	1	0	0	0	0	0	1	1	1	1	0	1
Piette, C. 2017 [37]	1	1	1	1	1	1	1	0	0	1	0	0	1	1	1	0	0	9
Portillo, I. 2018 [38]	1	1	1	1	9	1	0	1	1	1	0	0	1	1	1	1	0	1
Poskus, T. 2015 [39]	1	1	1	1	1	9	0	0	0	0	0	0	1	1	1	1	0	9
Remes-Troche, J. M. 2020 [40]	1	1	0	1	0	1	0	0	1	1	0	0	1	1	1	1	0	1
Rutka, M. 2020 [41]	1	1	0	1	1	1	0	1	1	0	0	0	1	1	1	1	0	1
Salimzadeh, H. 2017 [42]	1	1	0	1	1	1	0	1	1	1	0	0	1	1	1	1	0	1
Shahidi, N. 2016 [43]	1	1	1	1	9	1	0	1	1	0	0	0	1	1	1	1	0	1
Shim, J. I. 2010 [44]	1	1	1	1	9	9	0	1	1	1	0	0	1	1	1	1	0	9
Steele, R. J. 2013 [45]	1	1	1	1	1	9	0	1	1	0	0	0	1	1	1	0	0	1
Stegeman, I. 2015 [46]	1	1	0	1	1	1	1	0	1	0	0	0	1	1	1	1	0	9
Suchanek, S. 2014 [47]	1	1	1	1	9	9	0	0	1	0	0	0	1	1	1	1	0	9
Sultanian, R. 2020 [48]	1	1	0	1	1	1	0	1	1	1	0	0	1	1	1	1	0	1
Teixeira, C. R. 2017 [49]	1	1	0	1	9	0	0	0	0	1	0	0	1	1	1	1	9	1
Telford, J. 2016 [50]	1	1	0	1	1	1	0	0	0	1	0	0	1	1	1	1	0	1
Tepeš, B. 2014 [51]	1	1	0	1	1	1	0	1	1	0	0	0	1	1	1	0	0	9
Tepeš, B. 2017 [52]	1	1	1	1	1	1	0	1	1	1	0	0	1	1	1	1	0	9
Triantafillidis, J. K. 2010 [53]	1	1	0	1	1	0	0	0	0	0	0	0	1	1	1	0	9	9
Vitellius, C. 2019 [54]	1	1	1	1	1	0	0	1	1	0	0	0	1	1	1	1	9	9
Zorzi, M. 2006 [55]	1	1	1	1	1	0	1	0	0	0	0	0	1	1	0	0	9	9
Zorzi, M. 2007 [56]	1	1	1	1	1	0	1	0	0	0	0	0	1	1	0	0	9	9
Zorzi, M. 2008 [57]	1	1	1	1	1	0	1	0	0	0	0	0	1	1	0	0	9	9
Zorzi, M. 2009 [58]	1	1	1	1	1	0	1	0	0	0	0	0	1	1	0	0	9	9
Zorzi, M. 2010 [59]	1	1	1	1	1	0	1	0	0	0	0	0	1	1	0	0	9	9
Zorzi, M. 2011 [60]	1	1	1	1	1	0	1	0	0	0	0	0	1	1	0	0	9	9
Zorzi, M. 2015_1 [61]	1	1	1	1	1	0	1	0	0	0	0	0	1	1	0	0	9	9
Zorzi, M. 2015_2 [62]	1	1	1	1	0	0	0	1	1	1	0	0	1	1	1	1	0	1
Zorzi, M. 2018 [63]	1	1	1	1	1	0	1	1	1	1	0	0	1	1	1	1	0	9

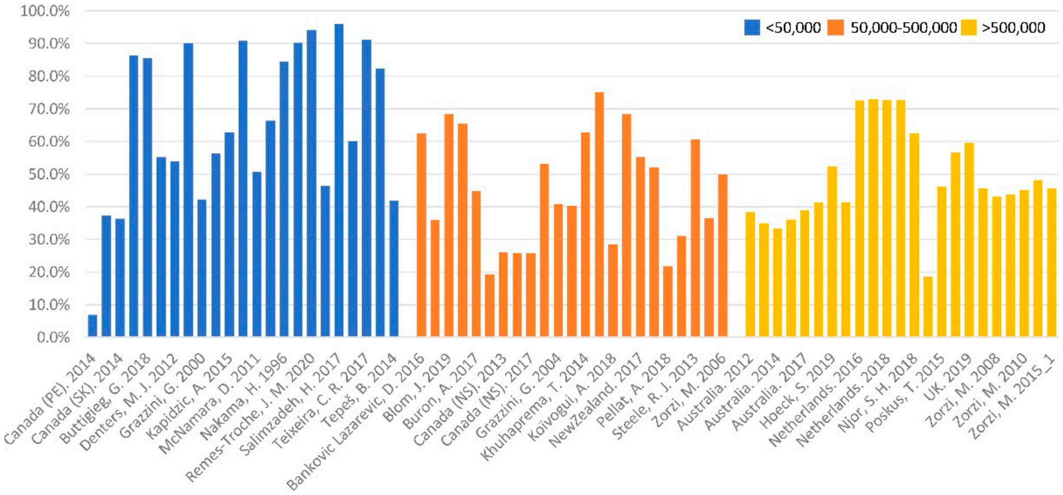

Figure S1. Participation rates by sample size.

Table References

1. Abu Hassan, M.R.; Leong, T.W.; Othman Andu, D.F.; Hat, H.; Nik Mustapha, N.R. Evaluation of a Colorectal Carcinoma Screening Program in Kota Setar and Kuala Muda Districts, Malaysia. *Asian Pac. J. Cancer Prev.* **2016**, *17*, 569–573.
2. Auge, J.M.; Pellise, M.; Escudero, J.M.; Hernandez, C.; Andreu, M.; Grau, J.; Buron, A.; López-Cerón, M.; Bessa, X.; Serradesanferm, A.; et al. Risk stratification for advanced colorectal neoplasia according to fecal hemoglobin concentration in a colorectal cancer screening program. *Gastroenterology* **2014**, *147*, 628–636.e1, doi:10.1053/j.gastro.2014.06.008.

3. Bankovic Lazarevic, D.; Krivokapic, Z.; Barisic, G.; Jovanovic, V.; Ilic, D.; Veljkovic, M. Organized colorectal cancer screening in Serbia - the first round within 2013–2014. *Vojnosanit. Pregl.* **2016**, *73*, 360–367.
4. Binefa, G.; Garcia, M.; Milà, N.; Fernández, E.; Rodríguez-Moranta, F.; Gonzalo, N.; Benito, L.; Clopés, A.; Guardiola, J.; Moreno, V. Colorectal Cancer Screening Programme in Spain: Results of Key Performance Indicators After Five Rounds (2000–2012). *Sci. Rep.* **2016**, *6*, 19532, doi:10.1038/srep19532.
5. Blom, J.; Löwbeer, C.; Elfström, K.M.; Sventelius, M.; Öhman, D.; Saraste, D.; Törnberg, S. Gender-specific cut-offs in colorectal cancer screening with FIT: Increased compliance and equal positivity rate. *J. Med. Screen.* **2019**, *26*, 92–97, doi:10.1177/0969141318804843.
6. Bujanda, L.; Sarasqueta, C.; Castells, A.; Pellisé, M.; Cubiella, J.; Gil, I.; Cosme, A.; Arana-Arri, E.; Mar, I.; Idigoras, I.; et al. Colorectal cancer in a second round after a negative faecal immunochemical test. *Eur. J. Gastroenterol. Hepatol.* **2015**, *27*, 813–818, doi:10.1097/MEG.0000000000000366.
7. Buron, A.; Auge, J.M.; Sala, M.; Román, M.; Castells, A.; Macià, F.; Comas, M.; Guiriguet, C.; Bessa, X.; Castells, X. Association between socioeconomic deprivation and colorectal cancer screening outcomes: Low uptake rates among the most and least deprived people. *PLoS One* **2017**, *12*, 1DUMMY, doi:10.1371/journal.pone.0179864.
8. Gabriella Buttigieg; Rachel Abela A Retrospective study on the National Colorectal Screening Programme: Analysis of participation and findings. *THESYNAPSE.net* **2018**, *17*, 6–8.
9. Chou, C.K.; Chen, S.L.S.; Yen, A.M.F.; Chiu, S.Y.H.; Fann, J.C.Y.; Chiu, H.M.; Chuang, S.L.; Chiang, T.H.; Wu, M.S.; Wu, C.Y.; et al. Outreach and inreach organized service screening programs for colorectal cancer. *PLoS One* **2016**, *11*, doi:10.1371/journal.pone.0155276.
10. Ciatto, S.; Martinelli, F.; Castiglione, G.; Mantellini, P.; Rubeca, T.; Grazzini, G.; Bonanomi, A.G.; Confortini, M.; Zappa, M. Association of FOBT-assessed faecal Hb content with colonic lesions detected in the Florence screening programme. *Br. J. Cancer* **2007**, *96*, 218–221, doi:10.1038/sj.bjc.6603534.
11. Clark, G.; Strachan, J.A.; Carey, F.A.; Godfrey, T.; Irvine, A.; McPherson, A.; Brand, J.; Anderson, A.S.; Fraser, C.G.; Steele, R.J.C. Transition to quantitative faecal immunochemical testing from guaiac faecal occult blood testing in a fully rolled-out population-based national bowel screening programme. *Gut* **2020**, doi:10.1136/gutjnl-2019–320297.
12. Crotta, S.; Castiglione, G.; Grazzini, G.; Valle, F.; Mosconi, S.; Rosset, R. Feasibility study of colorectal cancer screening by immunochemical faecal occult blood testing: Results in a northern Italian community. *Eur. J. Gastroenterol. Hepatol.* **2004**, *16*, 33–37, doi:10.1097/00042737–200401000-00006.
13. Denters, M.J.; Deutekom, M.; Bossuyt, P.M.; van Rijn, A.F.; Fockens, P.; Dekker, E. Involvement of previous non-participants cannot fully compensate for lower participation in a second round of FIT-screening. *Cancer Epidemiol.* **2013**, *37*, 330–335, doi:10.1016/j.canep.2013.01.007.
14. Fenocchi, E.; Martínez, L.; Tolve, J.; Montano, D.; Rondán, M.; Parra-Blanco, A.; Eishi, Y. Screening for colorectal cancer in Uruguay with an immunochemical faecal occult blood test. *Eur. J. Cancer Prev.* **2006**, *15*, 384–390, doi:10.1097/00008469–200610000-00002.
15. Grazzini, G.; Castiglione, G.; Isu, A.; Mantellini, P.; Rubeca, T.; Sani, C.; Turco, P.; Zappa, M. Colorectal cancer screening by fecal occult blood testing: Results of a population-based experience. *Tumori* **2000**, *86*, 384–388.
16. Grazzini, G.; Castiglione, G.; Ciabattoni, C.; Franceschini, F.; Giorgi, D.; Gozzi, S.; Mantellini, P.; Lopane, P.; Perco, M.; Rubeca, T.; et al. Colorectal cancer screening programme by faecal occult blood test in Tuscany: First round results. *Eur. J. Cancer Prev.* **2004**, *13*, 19–26, doi:10.1097/00008469–200402000-00004.
17. Grazzini, G.; Visioli, C.B.; Zorzi, M.; Ciatto, S.; Banovich, F.; Bonanomi, A.G.; Bortoli, A.; Castiglione, G.; Cazzola, L.; Confortini, M.; et al. Immunochemical faecal occult blood test: Number of samples and positivity cutoff. What is the best strategy for colorectal cancer screening? *Br. J. Cancer* **2009**, *100*, 259–265, doi:10.1038/sj.bjc.6604864.
18. Guo, F.; De Brabander, I.; Francart, J.; Candeur, M.; Polus, M.; Van Eycken, L.; Brenner, H. Benefits of switching from guaiac-based faecal occult blood to faecal immunochemical testing: experience from the Wallonia–Brussels colorectal cancer screening programme. *Br. J. Cancer* **2020**, *122*, 1109–1117, doi:10.1038/s41416–020-0754–5.
19. Hoeck, S.; van de Veerdonk, W.; De Brabander, I.; Kellen, E. Does the Flemish colorectal cancer screening programme reach equity in FIT uptake? *Eur J Public Heal.* **2019**, doi:10.1093/eurpub/ckz043.
20. Kapidzic, A.; van der Meulen, M.P.; Hol, L.; van Roon, A.H.C.; Looman, C.W.N.; Lansdorp-Vogelaar, I.; van Ballegooijen, M.; van Vuuren, A.J.; Reijerink, J.C.I.Y.; van Leerdam, M.E.; et al. Gender Differences in Fecal Immunochemical Test Performance for Early Detection of Colorectal Neoplasia. *Clin. Gastroenterol. Hepatol.* **2015**, *13*, 1464–1471.e4, doi:10.1016/j.cgh.2015.02.023.
21. Khuhaprema, T.; Sangrajrang, S.; Lalitwongsa, S.; Chokvanitphong, V.; Raunroadroong, T.; Ratanachu-Ek, T.; Muwonge, R.; Lucas, E.; Wild, C.; Sankaranarayanan, R. Organised colorectal cancer screening in Lampang Province, Thailand: preliminary results from a pilot implementation programme. *BMJ Open* **2014**, *4*, doi:10.1136/bmjopen-2013–003671.
22. Kim, D.H.; Cha, J.M.; Kwak, M.S.; Yoon, J.Y.; Cho, Y.H.; Jeon, J.W.; Shin, H.P.; Joo, K.R.; Lee, J.I. Quality Metrics of a Fecal Immunochemical Test-Based Colorectal Cancer Screening Program in Korea. *Gut Liver* **2018**, *12*, 183–189, doi:10.5009/gnl17030.
23. Koïvogui, A.; Le Mab, G.; Benamouzig, R. Detection of colorectal neoplasia in a cohort before and after the change of fecal occult blood test in a French colorectal cancer screening program. *Am. J. Gastroenterol.* **2018**, *113*, 1891–1899, doi:10.1038/s41395–018-0367–2.
24. Launoy, G.D.; Bertrand, H.J.; Berchi, C.; Talbourdet, V.Y.; Guizard, A.V.N.; Bouvier, V.M.; Caces, E.R. Evaluation of an immunochemical fecal occult blood test with automated reading in screening for colorectal cancer in a general average-risk population. *Int. J. Cancer* **2005**, *115*, 493–496, doi:10.1002/ijc.20921.
25. Lund, M.; Erichsen, R.; Valori, R.; Møller Jensen, T.; Helle Njor, S.; Laurberg, S.; Andersen, B. Data quality and colonoscopy performance indicators in the prevalent round of a FIT-based colorectal cancer screening program. *Scand. J. Gastroenterol.* **2019**, doi:10.1080/00365521.2019.1597158.
26. Manzano-Robleda, M.D.C.; Espinosa-Tamez, P.; Potter, M.B.; Lajous, M.; Van Loon, K.; Zhang, L.; Jimenez-Peña, A.; Sanchez Del Monte, J.; Mohar-Betancourt, A.; Hernández-Guerrero, A. Fecal immunological test results and diagnostic colonoscopy in a Mexican population at average risk for colorectal cancer. *Cancer Prev. Res. (Phila).* **2020**, doi:10.1158/1940–6207.CAPR-20–0076.
27. McNamara, D.; Qasim, A.; Lee, N.; Condon, C.; O'Morain, C. Round one of the Adelaide and Meath Hospital/Trinity College Colorectal Cancer Screening Programme: Programme report and analysis based on established international key performance indices. *Ir. J. Med. Sci.* **2011**, *180*, 549–552, doi:10.1007/s11845–010-0650–8.
28. Miuţescu, B.; Sporea, I.; Popescu, A.; Bota, S.; Iovănescu, D.; Burlea, A.; Mos, L.; Palaghia, M.; Vasilescu, A.; Miuţescu, E. Effectiveness of

the immunochemical fecal test (FIT) for detection of advanced adenomas in colorectal carcinoma screening in an asymptomatic population. *Rev. medico-chirurgicală a Soc. Medici şi Nat. din Iaşi* **2013**, *117*, 302–307.
29. Moss, S.; Mathews, C.; Day, T.J.; Smith, S.; Seaman, H.E.; Snowball, J.; Halloran, S.P. Increased uptake and improved outcomes of bowel cancer screening with a faecal immunochemical test: Results from a pilot study within the national screening programme in England. *Gut* **2017**, *66*, 1631–1644, doi:10.1136/gutjnl-2015–310691.
30. Nakama, H.; Kamijo, N.; Abdul Fattah, A.S.; Zhang, B. Validity of immunological faecal occult blood screening for colorectal cancer: a follow up study. *J. Med. Screen.* **1996**, *3*, 63–65.
31. Nakama, H.; Zhang, B.; Fukazawa, K.; Zhang, X. Comparisons of cancer detection rate and costs of one cancer detected among different age-cohorts in immunochemical occult blood screening. *J. Cancer Res. Clin. Oncol.* **2001**, *127*, 439–443.
32. Njor, S.H.; Friis-Hansen, L.; Andersen, B.; Søndergaard, B.; Linnemann, D.; Jørgensen, J.C.R.; Roikjær, O.; Rasmussen, M. Three years of colorectal cancer screening in Denmark. *Cancer Epidemiol.* **2018**, *57*, 39–44, doi:10.1016/j.canep.2018.09.003.
33. Okada, T.; Odagaki, T.; López-Köstner, F.; Zárate, A.J.; Ponce, A.; Kronberg, U.; Karelovic, S.; Flores, S.; Estela, R.; Ito, T.; et al. Colorectal cancer risk factors in asymptomatic Chilean population: a survey of international collaboration between Japan and Chile. *Eur. J. Cancer Prev.* **2019**, doi:10.1097/CEJ.0000000000000531.
34. Parente, F.; Boemo, C.; Ardizzoia, A.; Costa, M.; Carzaniga, P.; Ilardo, A.; Moretti, R.; Cremaschini, M.; Parente, E.M.; Pirola, M.E. Outcomes and cost evaluation of the first two rounds of a colorectal cancer screening program based on immunochemical fecal occult blood test in northern Italy. *Endoscopy* **2013**, *45*, 27–34, doi:10.1055/s-0032–1325800.
35. Park, M.J.; Choi, K.S.; Jun, J.K.; Lee, H.Y. Trends in the National Cancer Screening Program for colorectal cancer in the Republic of Korea, 2004–2009. *Asian Pac. J. Cancer Prev.* **2011**, *12*, 3489–3493.
36. Pellat, A.; Deyra, J.; Coriat, R.; Chaussade, S. Results of the national organised colorectal cancer screening program with FIT in Paris. *Sci Rep* **2018**, *8*, 4162, doi:10.1038/s41598–018-22481–9.
37. Piette, C.; Durand, G.; Bretagne, J.F.; Faivre, J. Additional mailing phase for FIT after a medical offer phase: The best way to improve compliance with colorectal cancer screening in France. *Dig. Liver Dis.* **2017**, *49*, 308–311, doi:10.1016/j.dld.2016.09.015.
38. Portillo, I.; Idigoras, I.; Bilbao, I.; Arana-Arri, E.; Fernández-Landa, M.J.; Hurtado, J.L.; Sarasaqueta, C.; Bujanda, L. Colorectal cancer screening program using FIT: Quality of colonoscopy varies according to hospital type. *Endosc. Int. Open* **2018**, *6*, E1149–E1156, doi:10.1055/a-0655–1987.
39. Poskus, T.; Strupas, K.; Mikalauskas, S.; Bitinaite, D.; Kavaliauskas, A.; Samalavicius, N.E.; Saladzinskas, Z. Initial results of the National Colorectal Cancer Screening Program in Lithuania. *Eur. J. Cancer Prev.* **2015**, *24*, 76–80, doi:10.1097/CEJ.0000000000000096.
40. Remes-Troche, J.M.; Hinojosa-Garza, G.; Espinosa-Tamez, P.; Meixueiro-Daza, A.; Grube-Pagola, P.; Van Loon, K.; Potter, M.B.; Lajous, M. Faecal immunochemical test-based colorectal cancer screening in Mexico: an initial experience. *Fam. Pract.* **2020**, *37*, 321–324, doi:10.1093/fampra/cmz078.
41. Rutka, M.; Bor, R.; Molnár, T.; Farkas, K.; Fábián, A.; Gy Rffy, M.; Bálint, A.; Milassin, Á.; Sz Cs, M.; Tiszlavicz, L.; et al. Efficacy of a population-based colorectal cancer screening pilot. *Turk J Med Sci* **2020**, *50*, 756–763, doi:10.3906/sag-1908–79.
42. Salimzadeh, H.; Bishehsari, F.; Sauvaget, C.; Amani, M.; Hamzehloo, G.; Nikfarjam, A.; Merat, S.; Delavari, A.; Malekzadeh, R. Feasibility of colon cancer screening by fecal immunochemical test in Iran. *Arch. Iran. Med.* **2017**, *20*, 726–733.
43. Shahidi, N.; Gentile, L.; Gondara, L.; Hamm, J.; McGahan, C.E.; Enns, R.; Telford, J. Correlating Quantitative Fecal Immunochemical Test Results with Neoplastic Findings on Colonoscopy in a Population-Based Colorectal Cancer Screening Program: A Prospective Study. *Can. J. Gastroenterol. Hepatol.* **2016**, *2016*, doi:10.1155/2016/4650471.
44. Shim, J.I.; Kim, Y.; Han, M.A.; Lee, H.Y.; Choi, K.S.; Jun, J.K.; Park, E.C. Results of colorectal cancer screening of the national cancer screening program in Korea, 2008. *Cancer Res Treat* **2010**, *42*, 191–198, doi:10.4143/crt.2010.42.4.191.
45. Steele, R.J.; McDonald, P.J.; Digby, J.; Brownlee, L.; Strachan, J.A.; Libby, G.; McClements, P.L.; Birrell, J.; Carey, F.A.; Diament, R.H.; et al. Clinical outcomes using a faecal immunochemical test for haemoglobin as a first-line test in a national programme constrained by colonoscopy capacity. *United Eur. Gastroenterol. J.* **2013**, *1*, 198–205, doi:10.1177/2050640613489281.
46. Stegeman, I.; van Doorn, S.C.; Mundt, M.W.; Mallant-Hent, R.C.; Bongers, E.; Elferink, M.A.G.; Fockens, P.; Stroobants, A.K.; Bossuyt, P.M.; Dekker, E. Participation, yield, and interval carcinomas in three rounds of biennial FIT-based colorectal cancer screening. *Cancer Epidemiol.* **2015**, *39*, 388–393, doi:10.1016/j.canep.2015.03.012.
47. Suchanek, S.; Majek, O.; Vojtechova, G.; Minarikova, P.; Rotnaglova, B.; Seifert, B.; Minarik, M.; Kozeny, P.; Dusek, L.; Zavoral, M. Colorectal cancer prevention in the Czech Republic: Time trends in performance indicators and current situation after 10 years of screening. *Eur. J. Cancer Prev.* **2014**, *23*, 18–26, doi:10.1097/CEJ.0b013e328364f203.
48. Sultanian, R.; Du, L.; Moysey, B.; Morse, A.; Veldhuyzen van Zanten, S.; Montano-Loza, A.J. The Impact of Transitioning From Guaiac-Fecal Occult Blood Testing to Fecal Immunochemical Testing in a Canadian Colon Cancer Screening Program. *J Can Assoc Gastroenterol* **2020**, *3*, 177–184, doi:10.1093/jcag/gwz009.
49. Teixeira, C.R.; Bonotto, M.L.; Lima, J.P.; Figueiredo, L.F.; Conrado, L.; Frasca, C. Clinical impact of the immunochemical fecal occult blood test for colorectal cancer screening in Brazil. *Ann. Gastroenterol.* **2017**, *30*, 442–445, doi:10.20524/aog.2017.0151.
50. Telford, J.; Gentile, L.; Gondara, L.; McGahan, C.; Coldman, A. Performance of a quantitative fecal immunochemical test in a colorectal cancer screening pilot program: a prospective cohort study. *C. Open* **2016**, *4*, E668-e673, doi:10.9778/cmajo.20160047.
51. Tepeš, B.; Štabuc, B.; Stefanovič, M.; Bračko, M.; Frkovič Grazio, S.; Novak Mlakar, D.; Maučec Zakotnik, J. Faecal immunochemical test-based colorectal cancer screening programme SVIT in Slovenia: Pilot phase. *Eur. J. Cancer Prev.* **2014**, *23*, 235–239, doi:10.1097/CEJ.0b013e3283651c9e.
52. Tepeš, B.; Bracko, M.; Novak Mlakar, D.; Stefanovic, M.; Stabuc, B.; Frkovic Grazio, S.; Maucec Zakotnik, J. Results of the FIT-based National Colorectal Cancer Screening Program in Slovenia. *J. Clin. Gastroenterol.* **2017**, *51*, e52–e59, doi:10.1097/MCG.0000000000000662.
53. Triantafillidis, J.K.; Kosmidis, P.A.; Papalois, A.; Merikas, E.; Cheracakis, P.; Govosdis, V.; Nicolakis, D.; Panteris, V.; Skourta, I.; Tountas, I.; et al. Screening programs for colorectal cancer in Greece: Results of two pilot studies conducted in March 2008 and 2009. *Ann. Gastroenterol.* **2010**, *23*, 42–47.
54. Vitellius, C.; Laly, M.; Banaszuk, A.S.; Deherce, I.; Cornet, N.; Bertrais, S.; Saulnier, P.; Caroli-Bosc, F.X. Contribution of the OC Sensor® immunoassay in comparison to the Hemoccult II® guaiac-test in organized colorectal cancer screening. *Eur. J. Epidemiol.* **2019**, *34*, 163–172,

doi:10.1007/s10654–018-0471-z.
55. Zorzi, M.; Grazzini, G.; Senore, C.; Vettorazzi, M. Screening for colorectal cancer in Italy: 2004 survey. *Epidemiol. Prev.* **2006**, *30*, 41–50.
56. Zorzi, M.; Barca, A.; Falcini, F.; Grazzini, G.; Pizzuti, R.; Ravaioli, A.; Sassoli de Bianchi, P.; Senore, C.; Sigillito, A.; Vettorazzi, M.; et al. Screening for colorectal cancer in Italy: 2005 survey. *Epidemiol. Prev.* **2007**, *31*, 49–60.
57. Zorzi, M.; Falcini, F.; Fedato, C.; Grazzini, G.; de' Bianchi, P.S.; Senore, C.; Vettorazzi, M.; Visioli, C.; Zappa, M. Screening for colorectal cancer in Italy: 2006 survey. *Epidemiol. Prev.* **2008**, *32*, 55–68.
58. Zorzi, M.; Fedato, C.; Naldoni, C.; Sassatelli, R.; Sassoli De' Bianchi, P.; Senore, C.; Visioli, C.B.; Cogo, C. Screening for colorectal cancer in Italy: 2007 survey. *Epidemiol. Prev.* **2009**, *33 Suppl 2*, 57–74.
59. Zorzi, M.; Baracco, S.; Fedato, C.; Grazzini, G.; Naldoni, C.; Sassoli dé Bianchi, P.; Senore, C.; Visioli, C.B.; Cogo, C. Screening for colorectal cancer in Italy: 2008 survey. *Epidemiol. Prev.* **2010**, *34*, 53–72.
60. Zorzi, M.; Baracco, S.; Fedato, C.; Grazzini, G.; Sassoli De' Bianchi, P.; Senore, C.; Visioli, C.B.; Cogo, C. Screening for colorectal cancer in Italy, 2009 survey. *Epidemiol. Prev.* **2011**, *35*, 55–77.
61. Zorzi, M.; Da Re, F.; Mantellini, P.; Naldoni, C.; Sassoli De'Bianchi, P.; Senore, C.; Turrin, A.; Visioli, C.B.; Zappa, M. Screening for colorectal cancer in Italy: 2011–2012 survey. *Epidemiol. Prev.* **2015**, *39*, 93–107.
62. Zorzi, M.; Senore, C.; Da Re, F.; Barca, A.; Bonelli, L.A.; Cannizzaro, R.; Fasoli, R.; Furia, L.D.; Giulio, E.D.; Mantellini, P.; et al. Quality of colonoscopy in an organised colorectal cancer screening programme with immunochemical faecal occult blood test: The EQuIPE study (Evaluating Quality Indicators of the Performance of Endoscopy). *Gut* **2015**, *64*, 1389–1396, doi:10.1136/gutjnl-2014–307954.
63. Zorzi, M.; Hassan, C.; Capodaglio, G.; Fedato, C.; Montaguti, A.; Turrin, A.; Rosano, A.; Monetti, D.; Stocco, C.; Baracco, S.; et al. Long-term performance of colorectal cancer screening programmes based on the faecal immunochemical test. *Gut* **2018**, *67*, 2124–2130, doi:10.1136/gutjnl-2017–314753.
64. Australian Institute of Health and Welfare and the Australian Government Department of Health and Ageing for the National Bowel Cancer Screening Program *National Bowel Cancer Screening Program Monitoring report 2006 - 2008*; Canberra, 2008;
65. Australian Institute of Health and Welfare and the Australian Government Department of Health and Ageing for the National Bowel Cancer Screening Program *National Bowel Cancer Screening Program Monitoring report 2008 - 2011*; Canberra, 2012;
66. Australian Institute of Health and Welfare *National Bowel Cancer Screening Program Monitoring report 2011–12*; Canberra, 2013;
67. Australian Institute of Health and Welfare *National Bowel Cancer Screening Program Monitoring report 2012–13*; Canberra, 2014;
68. Australian Institute of Health and Welfare *National Bowel Cancer Screening Program Monitoring report 2013–14*; Canberra, 2015;
69. Australian Institute of Health and Welfare *National Bowel Cancer Screening Program Monitoring report 2017*; Canberra, 2017;
70. Australian Institute of Health and Welfare *National Bowel Cancer Screening Program Monitoring report 2019*; Canberra, 2019;
71. Australian Institute of Health and Welfare *National Bowel Cancer Screening Program Monitoring report 2020*; Canberra, 2020;
72. Canadian Partnership Against Cancer *Colorectal Cancer Screening in Canada: Program Performance Results Report, January 2009 – December 2011* ; Toronto, 2013;
73. Canadian Partnership Against Cancer *Colorectal Cancer Screening in Canada: Monitoring & Evaluation of Quality Indicators – Results Report, January 2011 – December 2012*; Toronto, 2014;
74. Canadian Partnership Against Cancer *Colorectal Cancer Screening in Canada: Monitoring & Evaluation of Quality Indicators – Results Report, January 2013 – December 2014*; Toronto, 2017;
75. Non-Communicable Disease Branch, C. for H.P.D. of H.H.K.S.A.R.G. *Colorectal Cancer Screening Programme-Progress report of the screening outcome for participant enrolled between 28 September 2016 and 27 September 2019*; Hong Kong, 2019;
76. Cuid d'Fheidhmeannacht na Seirbhíse Sláinte. Part of the Health Service Executive (Ireland) *BowelScreen Programme Report Round One 2012–2015*; dublin, 2016;
77. Cuid d'Fheidhmeannacht na Seirbhíse Sláinte. Part of the Health Service Executive (Ireland) *BowelScreen Programme Report Round Two 2016–2017*; Dublin, 2019;
78. Nederlands kanker Instit; Uut *Monitoring and Evaluation of the Colorectal Cancer Screening Programme 2014*; 2015;
79. Nederlands kanker Instituut *Monitoring and Evaluation of the Colorectal Cancer Screening Programme 2015*; 2016;
80. Nederlands kanker Instituut *Monitoring and Evaluation of the Colorectal Cancer Screening Programme 2016*; 2017;
81. Nederlands kanker Instituut *Monitoring and Evaluation of the Colorectal Cancer Screening Programme 2017*; 2018;
82. National Institute for Public Health and the Environment (RIVM) *Monitoring and Evaluation of the Colorectal Cancer Screening Programme 2018*; 2019;
83. Ministry of Health (New Zealand) *Final Evaluation Report of the Bowel Screening Pilot Screening Rounds One and Two*; Wellington, 2016;
84. Quality Assurance Reference Centre, N.I.C.S.P.P.H.A. *Northern Ireland Bowel Cancer Screening Programme Inaugural Report*; BELFAST, 2014;
85. Information Services Division *Scottish Bowel Screening Programme Statistics For the two-year period of invitations between 1 November 2016 and 31 October 2018*; 2019;
